# First genomic analysis of a *Clostridium perfringens* strain carrying both the *cpe* and *netB* genes and the proposal of an amended toxin-based typing scheme

**DOI:** 10.3389/fmicb.2025.1580271

**Published:** 2025-05-19

**Authors:** Takashi Mada, Kenta Ochi, Mariko Okamoto, Daisuke Takamatsu

**Affiliations:** ^1^Division of Infectious Animal Disease Research, National Institute of Animal Health, National Agriculture and Food Research Organization, Tsukuba, Ibaraki, Japan; ^2^Ehime Prefectural Livestock Disease Diagnostic Center, Toon, Ehime, Japan; ^3^The United Graduate School of Veterinary Sciences, Gifu University, Gifu, Gifu, Japan; ^4^Joint Graduate School of Veterinary Sciences, Gifu University, Gifu, Gifu, Japan

**Keywords:** *Clostridium perfringens*, cattle, *cpe*, *netB*, type F, type G, untypable

## Abstract

*Clostridium perfringens* strains are classified into seven toxinotypes (A–G) based on the profiles of the six typing toxin genes. Among these toxins, NetB is known as an important virulence factor for necrotic enteritis in chickens, and its gene, *netB*, is present only in type G strains. CPE is the enterotoxin that causes food-borne affections in humans, and its gene, *cpe*, is carried by type F strains and occasionally by type C, D, and E strains. However, strains with both *netB* and *cpe* are extremely rare; thus, they are not assigned to either toxinotype under the current typing scheme. In 2022, a 69-month-old female Holstein cow in Japan died suddenly, and a *C. perfringens* strain (CP280) possessing both *netB* and *cpe* was isolated for the first time in Japan from the bovine intestinal contents. The CP280 genome was composed of one chromosome and six circular plasmids, and *netB* and *cpe* were carried on different plasmids, pCP280-82k and pCP280-55k, respectively. Multilocus sequence typing analysis assigned CP280 to ST21, and all other reported ST21 strains were type G strains. In the phylogenetic analysis using the genomes of 553 *C. perfringens* strains, CP280 was clustered into a group along with the type G strains from affected birds. The deduced amino acid sequences of NetB and CPE from CP280 were identical to those of NetB and CPE from avian necrotic enteritis cases and human food poisoning cases, respectively, implying the potential of CP280 to cause these diseases. The genetic relatedness of CP280 and type G strains strongly suggests that CP280 was originally type G with the *netB*-positive plasmid pCP280-82k and later acquired the *cpe*-positive plasmid pCP280-55k; therefore, CP280 should be treated as a type G strain. We propose to change the requirement for this toxinotype in the toxin-based typing scheme from *cpe*(−) to *cpe*(+/−).

## 1 Introduction

*Clostridium perfringens*, a Gram-positive spore-forming rod-shaped anaerobic bacteria, presents ubiquitously in the intestinal flora of animals and humans, food products or environment, however some strains of this bacterium can cause serious diseases, such as gas gangrene, foodborne gastroenteritis, antibiotic-associated diarrhea, and necrotic enteritis, in animals and humans (Kiu and Hall, 2018; Matches et al., 1974). The virulence of *C. perfringens* is attributed to the production of variety of toxins and extracellular enzymes (Kiu and Hall, 2018). *C. perfringens* strains had previously been classified into five toxinotypes (A, B, C, D, and E) based on the profiles of four typing toxin genes (*plc*/*cpa* [encoding alpha-toxin], *cpb* [beta-toxin], *etx* [epsilon-toxin], and *iap* and *ibp* [iota-toxin]). However, in 2018, with the addition of *cpe* encoding the *C. perfringens* pore-forming enterotoxin (CPE) and *netB* encoding necrotic enteritis toxin B-like toxin (NetB) as new typing toxin genes, two new toxinotypes, types F and G, were derived from type A, resulting in seven toxinotypes (A, B, C, D, E, F, and G) in this bacterium (Rood et al., 2018; Table 1). The *plc*/*cpa* gene is on the chromosome of all *C. perfringens* strains, *cpe* is on the chromosome or plasmids, and the other five genes, including *netB* are on plasmids (Li et al., 2013).

**Table 1:** Toxin-based typing scheme of *Clostridium perfringens*.

Toxinotype	Toxin (gene)					
	Alpha-toxin	Beta-toxin	Epsilon-toxin	Iota-toxin	Enterotoxin	NetB
	(*plc* or *cpa*)	(*cpb*)	(*etx*)	(*iap* and *ibp*)	(*cpe*)	(*netB*)
A	+	−	−	−	−	−
B	+	+	+	−	−	−
C	+	+	−	−	±	−
D	+	−	+	−	±	−
E	+	−	−	+	±	−
F	+	−	−	−	+	−
G	+	−	−	−	−	+
Untypable (CP280)	+	−	−	−	+	+

Among the seven toxinotypes, *C. perfringens* type F, which had been called as *cpe*-positive type A, can cause the gastrointestinal illnesses, including foodborne gastroenteritis, antibiotic-associated diarrhea, and sporadic diarrhea. Strains of this toxinotype possess *plc*/*cpa* and *cpe*, but not *cpb*, *etx*, *iap*, *ibp*, or *netB* (Rood et al., 2018). The *cpe* gene is not exclusively carried by type F strains, but can also be carried by type C, D, and E strains (Rood et al., 2018). The *cpe*-positive *C. perfringens* strains can be divided into two types according to the location of the *cpe* gene; that is, c-*cpe* strains that possess *cpe* on the chromosome and p-*cpe* strains that possess *cpe* on plasmids (Cornillot et al., 1995; Jaakkola et al., 2021). In the c-*cpe* strains, *cpe* is located on the transposable element Tn*5565* on the chromosome (Brynestad et al., 1997; Brynestad and Granum, 1999). p-*cpe* strains have the gene on an approximately 70–75-kbp plasmid, such as pCPF4969 (approximately 70 kbp) and pCPF5603 (approximately 75 kbp), and pCPF4969 is known to be horizontally transferable via conjugation (Miyamoto et al., 2006). Both types of *cpe*-positive strains are known to cause food poisoning.

*Clostridium perfringens* type G is equivalent of the previous *netB*-positive type A. It carries *plc*/*cpa* and *netB* (a pore-forming toxin gene belonging to the *Staphylococcus aureus* alpha-hemolysin-like beta-pore-forming toxin family), but not the other typing toxin genes, including *cpe* (Rood et al., 2018). Currently, type G is the only type that carries the *netB* gene, which is located on a conjugative plasmid of approximately 82 kbp (Parreira et al., 2012; Gohari et al., 2016). Alpha-toxin had once been considered a virulence factor of necrotic enteritis in chickens but was later revealed not to be essential for causing this disease (Al-Sheikhly and Truscott, 1977; Keyburn et al., 2006). On the other hand, NetB was revealed to have the most important role in the pathogenesis of necrotic enteritis in chickens, and the growth of type G strains and the production of NetB from the strains in the jejunum and ileum of chickens are considered crucial in its pathogenesis (Keyburn et al., 2008; Islam et al., 2021). Type G strains have been isolated almost exclusively from poultry, such as chickens; however, a type G strain isolated from the abscess of a three-year-old cow has also been reported (Smyth and Martin, 2010).

In 2022, a *C. perfringens* strain designated as CP280 was isolated from a Holstein cow that suddenly died in Japan. The isolate had *plc/cpa*, *cpe*, and *netB* and could not be classified into any toxinotypes because of the presence of both *cpe* and *netB* genes. Two *C. perfringens* strains with both *cpe* and *netB* genes have been isolated from layers in Germany (Gad et al., 2011). However, no detailed analysis of these strains, including genomic analysis, has been performed, and it is unknown how strains carrying these two genes arose. Furthermore, the isolation of *netB*-positive *C. perfringens* from cattle is extremely rare. Therefore, in the present study, we conducted the whole-genomic sequence analyses of the isolate and elucidated its virulence-related gene profile and genomic location of the *cpe* and *netB* genes to gain insight into how this strain has evolved and what potential risks this strain may pose to animals and humans. Furthermore, the phylogenetic relationships between CP280 and previously reported *C. perfringens* strains, including *cpe*-positive and *netB*-positive strains, were analyzed using the core genomes of these strains. Based on these results, we discussed the birth process of the *cpe* and *netB*-positive strain and proposed an amended toxin-based typing scheme.

## 2 Materials and methods

### 2.1 Case information

On the morning in August 2022, a 69-month-old Holstein female cow (8 months pregnant) kept on a farm in Japan died suddenly. The cow did not have any injuries or diarrhea, and had no previous clinical history, including mastitis. Necropsy revealed hemorrhages in the subcutis, diaphragm, and abomasum, and bleeding spots in the heart and greater omentum. Blood-like contents in the intestine and blood-like pericardial fluid were also observed. Microscope examination revealed serious histolysis of the mucous membranes in the alimentary canal (the abomasum, duodenum, cecum, and colon) and the suppurative and fibrinous serositis of the jejunum and cecum. The heart, lungs, liver, spleen, kidneys, intestinal contents, and pericardial and amniotic fluids were collected for bacteriological examinations. To search for causative organisms, the tissue samples were stamped or inoculated onto 5% sheep blood agar and modified GAM agar (Shimadzu Diagnostics, Corp., Tokyo, Japan) and incubated at 37°C for 24–48 h under anaerobic conditions. To quantify the number of *C. perfringens* cells in the intestinal contents, serially diluted samples were inoculated onto 5% egg yolk-supplemented kanamycin-containing CW agar (Shimadzu Diagnostics, Corp.), and yellowish-white *C. perfringens*-like colonies with white, opaque, and diffuse zones were counted after incubating the plates at 37°C for 24 h under anaerobic conditions. Colonies of Gram-positive and negative bacilli with positive catalase reactions grew from the pericardial fluid; however, because of the negligible number of colonies, we did not consider them to be the causative organisms in the death of the cow in this case. On the other hand, *C. perfringens* was detected in the small intestinal contents at a concentration of >1.0 × 10^6^ cells/g, and six *C. perfringens* strains (CP280–CP285) were isolated from this sample. No other bacterial pathogens were isolated from any of the other samples examined under the above culture conditions. In the present study, these six isolates from the intestinal contents were analyzed.

### 2.2 Genotyping

The toxinotypes of strains CP280–CP285 were identified by the PCR assays targeting six major toxin genes (*plc*/*cpa*, *cpb*, *etx*, *ia*p/*ibp*, *cpe*, and *netB*) using the primers described in a previous study (Rood et al., 2018). PCR was performed in a final reaction volume of 20 μL containing 1 × Takara Ex Premier™ DNA Polymerase (Takara Bio Inc., Kusatsu, Japan), 0.3 μM of each primer, and 1 μL of template DNA extracted by InstaGene Matrix (Bio-Rad Laboratories, Inc., Hercules, CA, United States) according to the manufacturer’s instructions. Reaction conditions consisted of an initial denaturation step at 94°C for 1 min, followed by 30 cycles of denaturation at 98°C for 10 s, annealing at 55°C for 15 s, and extension at 68°C for 1 min, followed by a final extension at 68°C for 5 min.

Multilocus sequence typing (MLST) of CP280–CP285 was performed by sequencing eight genes (*colA*, *groEL*, *gyrB, nadA*, *pgk*, *plc*, *sigK*, and *sodA*) as described previously (Deguchi et al., 2009) using DNA samples extracted with InstaGene Matrix (Bio-Rad). Sequencing was performed using the BigDye™ Terminator v3.1 Cycle Sequencing Kit (Applied Biosystems, Foster City, CA, United States), BigDye™ XTerminator Purification Kit (Applied Biosystems), and a 3500*xl* Genetic Analyzer (Applied Biosystems). The sequence types (STs) of the isolates were assigned by the submission of the data to the *C. perfringens* MLST database.^1^

### 2.3 Extraction of bacterial DNA for a genome sequence analysis

According to the methods described by Okamoto et al. (2021), the genomic DNA of CP280 was manually extracted from the strain cultured on modified GAM agar (Nissui Pharmaceutical Co.) supplemented with 5% sheep blood at 37°C for 24 h under anaerobic conditions. Briefly, after colonies of CP280 were suspended in TE (10 mM Tris–HCl [pH 8.0] and 1 mM EDTA [pH 8.0]), the mixture was treated with lysozyme and mutanolysin and lysed with 10% sodium dodecyl sulfate. Then, the lysate was treated with phenol, phenol-chloroform-isoamyl alcohol (25:24:1) (PCI), and chloroform once, thrice, and twice, respectively. Nucleic acids were precipitated, washed with ethanol, and resuspended in sterile H_2_O. After the RNase treatment and additional extraction with PCI and chloroform, bacterial DNA was precipitated again, rinsed with ethanol, and dissolved in 10 mM Tris–HCl (pH 8.5).

### 2.4 Genome sequencing, *de novo* assembly, and gene annotation

The whole-genome sequencing data of CP280 were obtained using the MinION platform (Oxford Nanopore Technologies, Plc., Oxford Science Park, OX, United Kingdom) and the DNBSEQ-G400 platform with paired-end 2 × 150-bp reads (BGI Genomic Co., Ltd., Shenzhen, China). DNA samples were prepared using the Rapid Barcoding Kit, SQK-RBK004 (Oxford Nanopore Technologies) (for the MinION platform), BGI Optimal DNA Library Kit, and MGI PE150 Kit (BGI Genomic Co., Ltd.) (for the DNBSEQ-G400 platform). After basecalling with high accuracy mode and dna_r9.4.1_450bps model and barcoding using Guppy (v. 6.4.6) (available from ONT community site: https://community.nanoporetech.com), MinION long reads with length less than 1,000 bp and quality lower than 10 were trimmed through NanoFilt (v. 2.8.0) (De Coster et al., 2018), and the quality of the remaining data was assessed using NanoStat (v. 1.6.0) (De Coster et al., 2018) and NanoPlot (v. 1.42.0) (De Coster et al., 2018). For DNBSEQ short reads, low-quality sequences and adapter sequences were trimmed using SOAPnuke developed by BGI with filters parameters “-n 0.01 -l 20 -q 0.4 –rmdup –adaMis 3 –outQualSys 1 –minReadLen 150” (v. 2.2.2) (Chen et al., 2018). Both the long and short reads were employed for *de novo* hybrid assembly using Unicycler (v. 0.5.0) (Wick et al., 2017) with default parameters. In the *de novo* hybrid assembly, the reads coverages per genome sizes was approximately ×1637.59. After all contigs were acknowledged as “circular topology” in the Unicycler output information, assembly accuracy was verified by mapping BGI short reads through Pilon (v. 1.24) (Walker et al., 2014). This mapping was repeated four times using default parameters until no nucleotide sequence revisions were detected in the output file. Each circular contig was rotated or changed to a reverse complementary sequence using SeqKit (v. 0.16.1) (commands: [seqkit restart -i] and [seqkit seq -pr]), as needed (Shen et al., 2016). Gene annotation was conducted using DFAST-core (Tanizawa et al., 2018). Genome sequences were deposited in the DDBJ/GenBank/EMBL databases under BioProject accession number PRJDB16159, BioSample accession number SAMD00628698, and DRA accession number DRA016639. Complete genome sequence data of CP280 were registered in the above database with accession numbers AP028647-AP028653.

### 2.5 Genome analysis

A total of 585 “*C. perfringens*” RefSeq genomes were available from the NCBI RefSeq database^2^ as of May 31st, 2023. The RefSeq genomes and CP280 genome were annotated by DFAST-core (v. 1.2.21). Because “Cpa/Plc” (WP_011590041.1) and/or “ColA” (BAB79879.1), whose genes are used for the MLST analysis of *C. perfringens*, were not found in 33 of the 585 RefSeq genomes by BLASTn (v. 2.11.0+) (Camacho et al., 2009), these 33 genomes were excluded from further analysis. The remaining 552 genomes and CP280 genome were confirmed to have average nucleotide identity (ANI) values greater than 95% for each other (i.e., to be *C. perfringens*) (Goris et al., 2007) using Mummer (Kurtz et al., 2004) under the Pyani (v. 0.2.12) (Pritchard et al., 2016) and used in the genome analyses (Supplementary Table 1).

For phylogenetic analyses, core genes (identity ≥ 90%) of the 553 *C. perfringens* strains were selected using Roary (v. 3.13.0) (Page et al., 2015). The single nucleotide polymorphisms (SNPs) in the *C. perfringens* population were extracted from the alignment of the core genes using SNP-sites (Page et al., 2016). The evolutionary history of the 553 sequences was inferred using the Neighbor-Joining method (Saito and Nei, 1987) with 1,000 bootstrap replicates (Felsenstein, 1985). The tree was drawn to scale, with branch lengths in the same units as those of the evolutionary distances used to infer the phylogenetic tree. The evolutionary distances were computed using the Maximum Composite Likelihood method (Tamura et al., 2004) and are in the units of the number of base substitutions per site. All ambiguous positions were removed for each sequence pair (pairwise deletion option). There were a total of 109,350 positions in the final dataset. Evolutionary analyses were conducted using MEGA11 (Tamura et al., 2021; Stecher et al., 2020). The phylogenetic tree was visualized using Interactive Tree of Life (iTOL) (v. 5) (Letunic and Bork, 2021), and the analyzed strains were divided into clades (BAPS groups) using RhierBAPS (Tonkin-Hill et al., 2018).

To further investigate the phylogenetic relationship between strains in BAPS group 1, core genes (identity ≥90%) of 117 *C. perfringens* strains in the group including strain CP280 were selected using Roary (v. 3.13.0) (Page et al., 2015). The SNPs in the *C. perfringens* population were extracted from the alignment of the core genes using SNP-sites (Page et al., 2016). The evolutionary history of the 117 sequences containing data for a total of 58,394 positions was inferred as described above.

A total of 553 genomes were also searched for the 36 virulence-related genes of *C. perfringens* (toxin, toxin-like protein, and putative virulence-associated enzyme genes analyzed in the previous study by Mada et al., 2023; Supplementary Table 2). A BLAST nucleotide database of the *C. perfringens* genomes was created using Makeblastdb (v. 2.11.0+) (Camacho et al., 2009), and tBLASTn (v. 2.11.0+) (Camacho et al., 2009) was used to identify the 36 virulence-related genes. In the present study, we regarded genes as virulence-related genes when the deduced protein sequences exhibited both ≥90% identity and ≥80% coverage with the reference virulence-related gene products (Supplementary Table 2). Except for CP280, the analyzed *C. perfringens* strains were classified into one of the seven toxinotypes (A–G) according to the presence or absence of the major toxin genes of *C. perfringens*: *plc/cpa*, *cpb*, *etx*, *iap/ibp*, *cpe*, and *netB* (Rood et al., 2018).

### 2.6 Comparison of plasmids and virulence-related genes between CP280 and other type F and G strains

The complete nucleotide sequences of *cpe*-positive plasmids from the type F strains and *netB*-positive plasmids from the type G strains were downloaded from the NCBI database and used for comparative analyses. To compare the genetic organization of plasmids between CP280 and other type F and G strains, clinker (Gilchrist and Chooi, 2021) was used in the CAGECAT (Van den Belt et al., 2023).^3^

The deduced amino acid sequence of NetB of CP280 was aligned and compared with NetB of 51 type G strains isolated from necrotic enteritis cases in chickens using MEGA 11 (Tamura et al., 2021; Stecher et al., 2020). Likewise, the deduced amino acid sequence of CPE of CP280 was aligned and compared with CPE of 25 type E and F strain(s) isolated from food poisoning cases in humans and a type E strain (CP PB-1) with the functional *cpe* gene (Miyamoto et al., 2011; Supplementary Table 1).

## 3 Results

### 3.1 Genotyping of *Clostridium perfringens* strains

Toxinotyping PCR revealed that *C. perfringens* CP280 was positive for *plc*/*cpa*, *cpe*, and *netB* and negative for *cpb*, *etx*, and *iap*/*ibp*. Following the current toxinotyping scheme (Table 1), this toxin gene profile (*plc*/*cpa*+, *cpb*-, *etx-*, *iap*/*ibp*-, *cpe*+, and *netB*+) did not fit either type; therefore, CP280 was determined to be untypable. According to the MLST analysis, CP280 was assigned to ST21 (allele profile, *colA*/*groEL*/*sodA*/*plc*/*gyrB*/*sigK*/*pgk*/*nadA* = 3/1/3/4/3/2/1/1) (Supplementary Table 3). In the PubMLST database^4^ (Jolley et al., 2018), there were five strains (98.78718-2 and C26 from Denmark, 2016TE7641_69 from Italy, and CP201-2017 and CP238-2017 from Japan) have been registered as ST21 (Keyburn et al., 2010; Lacey et al., 2018; Nauerby et al., 2003; Profeta et al., 2020; Ronco et al., 2017; Supplementary Table 4). Interestingly, all the five ST21 strains on the database were classified as type G because of the presence of the *plc*/*cpa* and *netB* genes, suggesting that CP280 is genetically closely related to type G strains.

Strains CP281-CP285 carried the *plc*/*cpa* gene only among the major toxin genes and were thus classified as type A following the current toxinotyping scheme (Table 1). Strains CP281 and CP284 were assigned to ST32 (allele profile, 22/5/17/21/15/16/7/19). Strains CP282, CP283 and CP285 were assigned as novel STs, CP282 and CP283 as ST719 (allele profile, 6/162/5/3/2/5/4/24) and CP285 as ST720 (allele profile, 25/4/46/148/111/27/22/41) (Supplementary Table 3). The allele profiles of these STs were quite different from that of ST21, suggesting that these strains are genetically distantly related to CP280.

### 3.2 Genome information of *Clostridium perfringens* CP280

The genome of strain CP280 was approximately 3.7M bp in size and consisted of one chromosome (3,445,394 bp: accession number AP028647) and six plasmids (pCP280-82k [82,749 bp], pCP280-76k [76,703 bp], pCP280-55k [55,009 bp], pCP280-38k [38,923 bp], pCP280-4k [4,145 bp], and pCP280-3k [3,200 bp]: accession number AP028648-AP028653) (Table 2). The topology of all chromosome and plasmid sequences was identified as circular from Unicycler (v 0.5.0).

**Table 2:** Genome characteristics of *Clostridium perfringens* CP280.

Constituent		Topology	Size (bp)	GC (%)	CDSs^a^	rRNA	tRNA	CRISPRs^b^	Genes specially mentioned in this study	Accession number
Chromosome		Circular	3,445,394	28.4	3,135	30	96	0	*plc/cpa*, *pfoA*, *nanH*, *nanI*, *nanJ*, *nanK*, *nagH*, *nagI*, *nagJ*, *nagK*, *colA*, *ccp/cloSI*	AP028647
Plasmid	pCP280-82k	Circular	82,749	25.7	80	0	0	0	*netB*	AP028648
Plasmid	pCP280-76k	Circular	76,703	26.1	81	0	0	0	*cpb2*	AP028649
Plasmid	pCP280-55k	Circular	55,009	26.6	64	0	0	0	*cpe, cpb2*	AP028650
Plasmid	pCP280-38k	Circular	38,923	26.4	48	0	0	0	None	AP028651
Plasmid	pCP280-4k	Circular	4,145	22.9	5	0	0	0	None	AP028652
Plasmid	pCP280-3k	Circular	3,200	23.2	3	0	0	0	None	AP028653
Total		NA^c^	3,706,123	28.2	3,416	30	96	0	NA^c^	NA^c^

^a^CDSs, coding sequences. ^b^CRISPRs, Clustered regularly interspaced short palindromic repeats. ^c^NA, not applicable.

Among the 36 virulence-related genes investigated in the present study (Supplementary Table 2), CP280 had 12 virulence-related genes on its chromosome (*plc*/*cpa*, *pfoA*, *nanH*, *nanI*, *nanJ*, *nanK*, *nagH*, *nagI*, *nagJ*, *nagK*, *colA*, and *ccp*/*cloSI*) and 4 virulence-related genes on plasmids (*netB* on pCP280-82k, *cpb2* on pCP280-76k, and *cpe* and *cpb2* on pCP280-55k) (Table 2; Supplementary Table 1).

### 3.3 Phylogenetic position of CP280 in the *Clostridium perfringens* population

**Figure 1:**
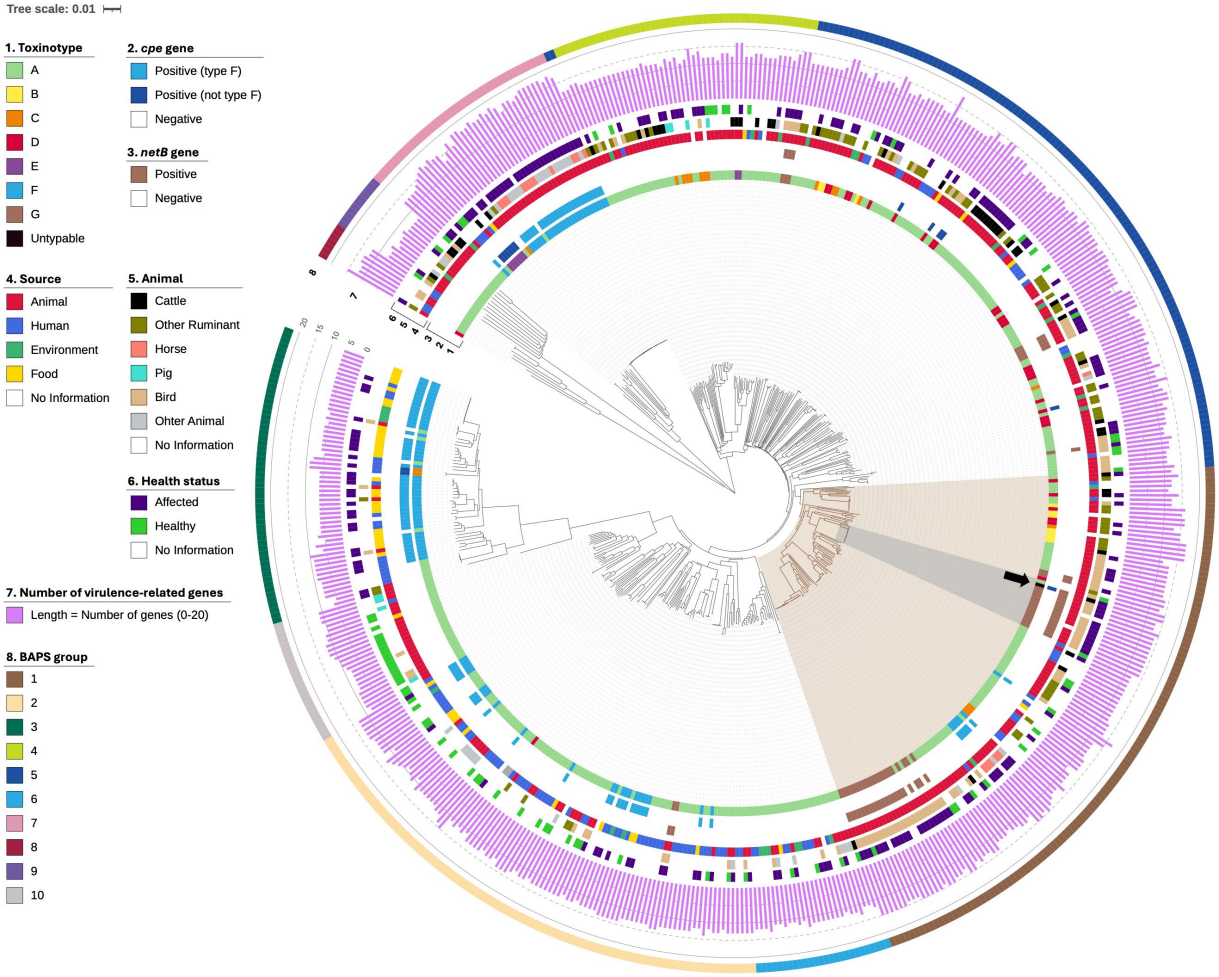
Core gene-based Neighbor-Joining SNPs tree of 553 *C. perfringens* strains. The 553 strains were divided into 10 BAPS groups (1–10) using rhierBAPS. From the inside, colored concentric rings represent the toxinotype, *cpe* gene possession, *netB* gene possession, isolation source, details of the source animal, health status of the source, number of virulence-related genes in each strain, and the BAPS group. The scale bar represents 0.01 substitutions per nucleotide. CP280 is indicated by a black arrow.

Based on the gene profiles of the six major toxin genes, the 553 *C. perfringens* strains analyzed in the present study were divided into 339 type A, 10 type B, 19 type C, 31 type D, 8 type E, 100 type F, 45 type G, and one untypable strain(s) (Figure 1; Supplementary Table 1). Among the 553 strains, only CP280 was untypable.

**Figure 2:**
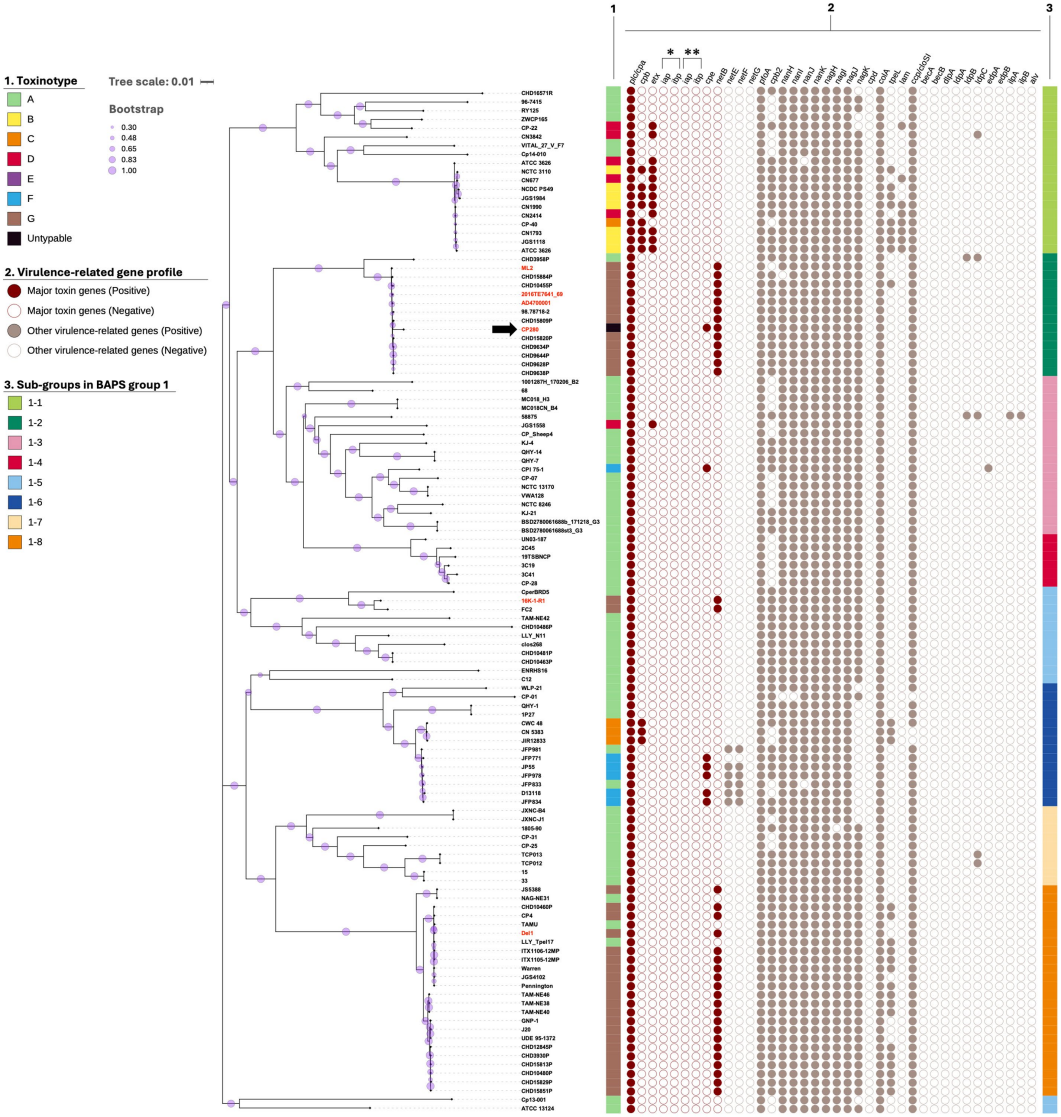
Core gene-based Neighbor-Joining SNPs tree of BAPS group 1 *C. perfringens* strains (left side) and 36 virulence-related gene profiles of the strains (right side). BAPS group 1 strains were divided into eight BAPS sub-groups (1-1–1-8) using rhierBAPS. Bootstrap values are indicated by the size of purple circles on the branches. The scale bar represents 0.01 substitutions per nucleotide. CP280 is indicated by a black arrow, and the type G strains, whose *netB*-positive plasmids were compared in the present study, are indicated in red letters. The possession of virulence-related genes in each strain was determined by ≥90% identity and ≥80% coverage with the reference virulence-related gene sequences. Two types of *iap*/*ibp* genes encoding a508.17-type (*) and JGS1987-type (**) iota-toxins were defined by Mada et al. (2023).

The phylogenetic tree revealed the 1,060 core genes in the 29,598 total genes and the presence of 10 BAPS groups (1–10) in the 553 *C. perfringens* strains (Figure 1; Supplementary Table 1). One hundred seventeen strains in BAPS group 1 shared 1,763 core genes in the 13,028 total genes and were further classified into eight BAPS sub-groups (1-1–1-8) (Figures 1, 2; Supplementary Table 1). Most of the type G strains belonged to BAPS sub-groups 1-2 and 1-8, and one of the differences between the two sub-groups was the possession rate of *tpeL*, with those of sub-group 1-2 and 1-8 being 8.33% (1/12 strains) and 71.43% (15/21 strains), respectively (Figure 2). Type F strains were assigned to four BAPS groups (6, 23, 45, and 26 strains to BAPS groups 1, 2, 3, and 7, respectively). Type F strains in BAPS groups 1 and 7 isolated mostly from affected animals possessed more virulence-related genes of *C. perfringens* (approximately 15 [group 1] and 17 [group 7] genes on average) than BAPS groups 2 and 3 strains isolated mainly from affected humans, food, and environmental samples (approximately 13 [group 2] and 7 [group 3] genes on average), showing the diversity of virulence-related gene profiles among type F strains. As mentioned above, CP280 had 16 virulence-related genes (including two copies of *cpb2*), which was greater than the number of virulence-related genes of strains in BAPS groups 2 and 3 from affected humans and food. In contrast, the virulence-related gene profile of CP280 was similar to those of a515.17 (a type E strain in BAPS group 7 isolated from a human food poisoning case) and CP PB-1 (a type E strain in BAPS group 7 reported to have the functional *cpe* gene) (Figure 1; Supplementary Table 1).

Interestingly, CP280 belonged to BAPS sub-group 1-2 [Figures 1, 2 (CP280 strain is indicated by the black arrow)]. This sub-group consisted of 14 strains, most of which were type G strains isolated from the affected birds, further supporting the close genetic relationship between CP280 and type G strains. In contrast to the type F strains, the type G strains belonging to any of BAPS groups 1, 2, 4, and 5 had similar virulence-related gene profiles to each other, consisting of 13–15 genes, and CP280 had almost the same virulence-related gene profile as these strains (Figures 1, 2; Supplementary Table 1).

### 3.4 Comparison of plasmids between CP280 and type F and G strains

**Figure 3:**
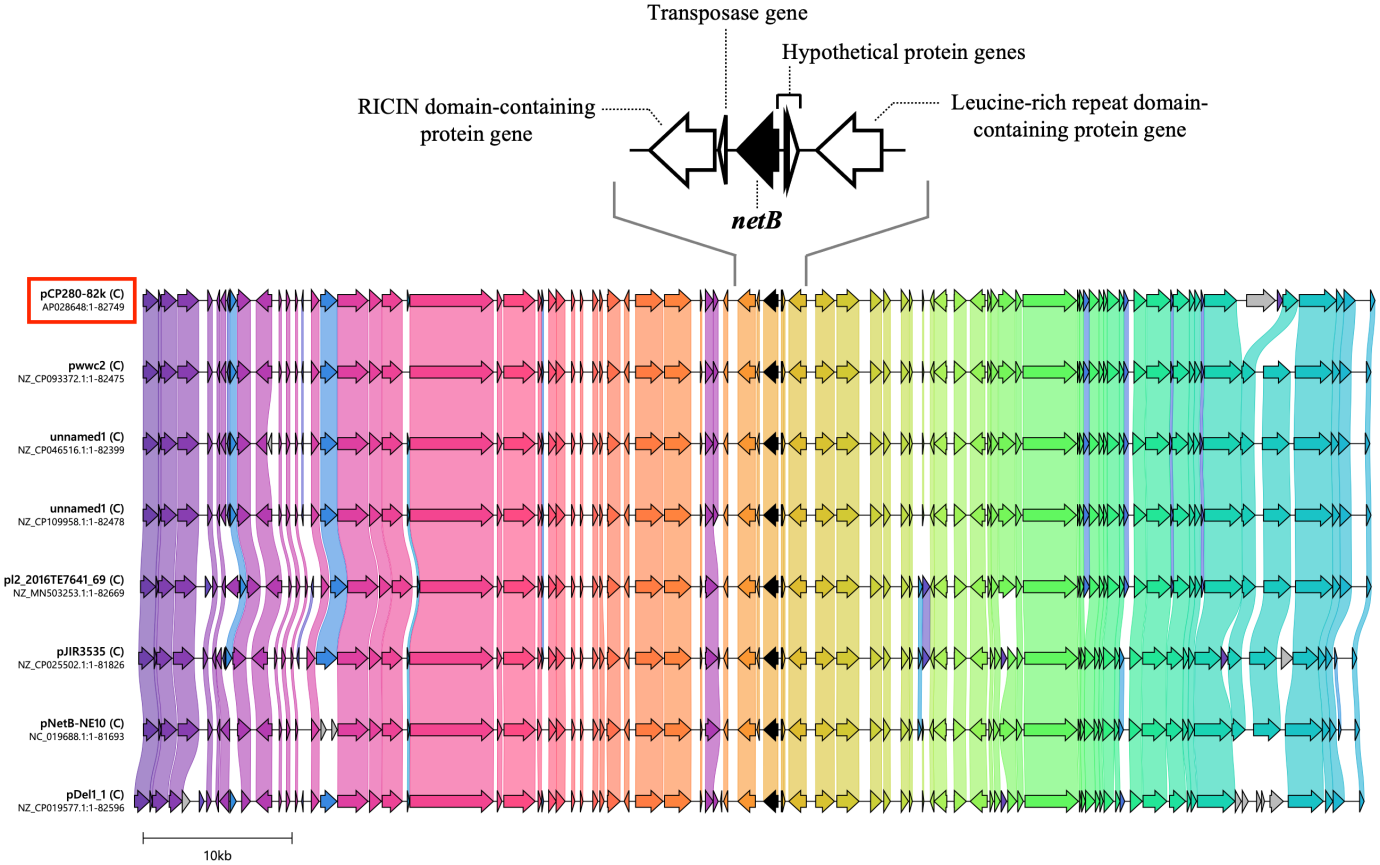
Comparison of the genetic organization of *netB*-positive plasmids between CP280 and type G strains. The nucleotide sequences of seven *netB*-positive plasmids of type G strains were downloaded from the NCBI database, and the genetic organization of the plasmids was compared with that of pCP280-82k from strain CP280 using clinker under CAGECAT. The arrows and arrowheads represent genes. *netB* is indicated by black arrows. The genetic organization of the eight plasmids was almost identical. Genes with 50% or more nucleotide sequence identity are connected to each other by colored bands. (C) described next to the strains’ name indicates the topology “Circular”.

All the eight *netB*-positive plasmids analyzed (pCP280-82k and seven from type G strains in the NCBI database) were circular in topology and of approximately 82 kbp. Their characteristics (plasmid size, GC content, coding ratio, gap ratio, and number of CDSs, rRNA, tRNA, and CRISPRs) and genetic organization (gene types and arrangement) were almost identical (Figure 3; Table 3).

**Table 3:** *netB*-positive plasmids from *Clostridium perfringens* CP280 and other type G strains.

Strain	Plasmid name	Topology	Size (bp)	GC (%)	CDSs^b^	rRNA	tRNA	CRISPRs^c^	Accession number
CP280	pCP280-82k	Circular	82,749	25.7	80	0	0	0	AP028648
2016TE7641_69	pl2_2016TE7641_69	Circular	82,669	25.7	81	0	0	0	NZ_MN503253.1
Del1	pDel1_1	Circular	82,596	25.7	82	0	0	0	NZ_CP019577.1
AD4700001	unnamed1	Circular	82,478	25.6	79	0	0	0	NZ_CP109958.1
ML2	pwwc2	Circular	82,475	25.7	79	0	0	0	NZ_CP093372.1
16K-1-R1	unnamed1	Circular	82,399	25.7	80	0	0	0	NZ_CP046516.1
EHE-NE18	pJIR3535	Circular	81,826	25.7	83	0	0	0	NZ_CP025502.1
NE_10^a^	pNetB-NE10	Circular	81,693	25.7	78	0	0	0	NC_019688.1

^a^In strain NE_10, only the plasmid sequence is available in the NCBI database. ^b^CDSs, coding sequences. ^c^CRISPRs, Clustered regularly interspaced short palindromic repeats.

**Figure 4:**
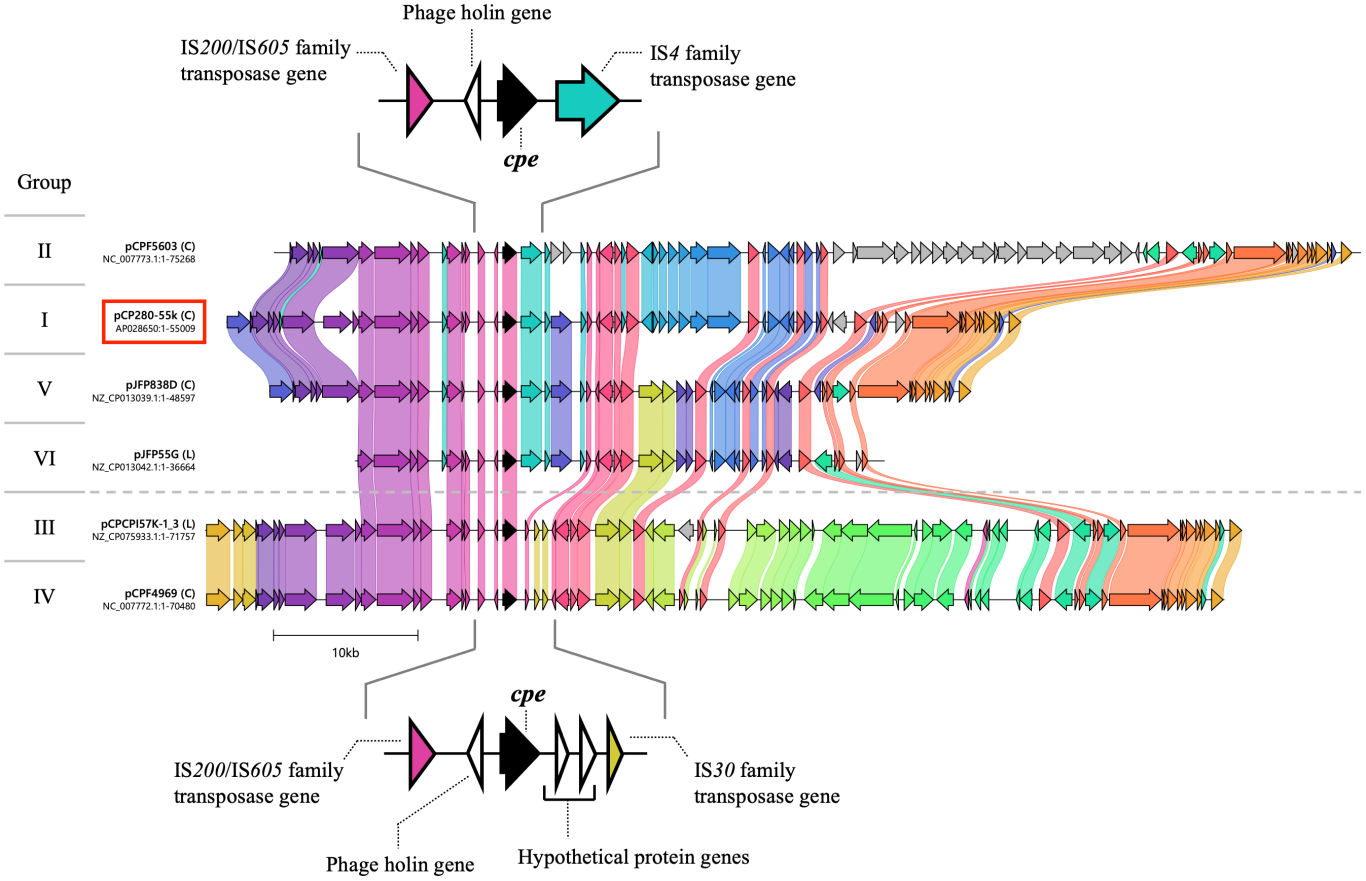
Comparison of the genetic organization of *cpe*-positive plasmids between CP280 and type F strains. The nucleotide sequences of 19 *cpe*-positive plasmids of type F strains were downloaded from the NCBI database, and the genetic organization of the plasmids was compared with that of pCP280-55k from strain CP280 using clinker under CAGECAT. The arrows and arrowheads represent genes. *cpe* is indicated by black arrows. Genes with 50% or more nucleotide sequence identity are connected to each other by colored bands. The 20 plasmids were classified into six groups (I–VI) based on their genetic organization, plasmid length, and types of transposase genes flanking the *cpe* gene. pCP280-55k was the only member of group I, while the other 19 plasmids were classified in groups II–VI (seven in group II, three in group III, six in group IV, two in group V, and one in group VI). This figure shows the genetic organization of the representative plasmids in each group. (C) and (L) described next to the strains’ name indicate the topology “Circular” and “Linear”, respectively.

Compared to *net*-positive plasmids, *cpe*-positive plasmids are structurally diverse, and the nucleotide sequences of 20 *cpe*-positive plasmids (pCP280-55k and 19 from the NCBI database) were divided into six groups (I–VI) based on their size and genetic organization. The largest were approximately 75 kbp plasmids in group II, and the smallest was the approximately 37 kbp plasmid in group VI (Figure 4; Table 4). pCP280-55k, with approximately 55 kbp, was the only member of group I. Groups II–V contained multiple plasmids, and the nucleotide sequences of the plasmids in the same group were largely identical. These six groups can be divided into two types by focusing on the structure around the *cpe* gene. In group I, II, V, and VI plasmids, the *cpe* and phage holin genes were located between an IS*200*/IS*605* family and an IS*4* family transposase genes, whereas group III and IV plasmids contained the *cpe*, phage holin, and two hypothetical genes between an IS*200*/IS*605* family and an IS*30* family transposase genes (Figure 4).

**Table 4:** *cpe*-positive plasmids from *Clostridium perfringens* CP280 and other type F strains.

Strain	Plasmid name	Topology	Size (bp)/Group		GC (%)	CDSs^a^	rRNA	tRNA	CRISPRs^b^	Accession number
CP280	pCP280-55k	Circular	55,009	I	26.6	64	0	0	0	AP028650
F5603	pCPF5603	Circular	75,268	II	25.5	82	0	0	0	NC_007773.1
721/84	pCP721-84_1	Linear	75,262		25.5	81	0	0	0	NZ_CP075954.1
AAD1527a	pCPAAD1527_2	Linear	75,262		25.5	81	0	0	0	NZ_CP076002.1
CPI103K-3	pCPCPI103K-3_1	Linear	75,262		25.5	81	0	0	0	NZ_CP075989.1
CPLi3-1	pCPCPLi3-1_1	Linear	75,262		25.5	82	0	0	0	NZ_CP075918.1
CPI63K-r5	pCPCPI63K-r5_1	Linear	75,258		25.5	80	0	0	0	NZ_CP075928.1
CPI75-1	pCPCPI75-1_1	Linear	75,068		25.5	80	0	0	0	NZ_CP075923.1
C216	pCPC216_1	Linear	71,757	III	26.7	72	0	0	0	NZ_CP075974.1
CPI39-1a	pCPCPI39-1_1	Linear	71,757		26.7	71	0	0	0	NZ_CP075942.1
CPI57K-1	pCPCPI57K-1_3	Linear	71,757		26.7	71	0	0	0	NZ_CP075933.1
F4969	pCPF4969	Circular	70,480	IV	26.6	70	0	0	0	NC_007772.1
CPI18-1b	pCPCPI18-1_1	Linear	70,476		26.6	70	0	0	0	NZ_CP075982.1
CPLi6-1	pCPCPLi6-1_1	Linear	70,476		26.6	70	0	0	0	NZ_CP075913.1
CPM77b	pCPCPM77b_2	Linear	70,476		26.6	70	0	0	0	NZ_CP075910.1
AAD1900a	pCPAAD1900a_1	Linear	70,475		26.6	71	0	0	0	NZ_CP075997.1
C269	pCPC269_1	Linear	70,475		26.6	70	0	0	0	NZ_CP075971.1
JP838	pJFP838D	Circular	48,597	V	26.6	56	0	0	0	NZ_CP013039.1
CPI53k-r1	pCPCPI53k-r1_3	Linear	48,590		26.6	56	0	0	0	NZ_CP075937.1
JP55	pJFP55G	Linear	36,664	VI	26.2	41	0	0	0	NZ_CP013042.1

^a^CDSs, coding sequences. ^b^CRISPRs, Clustered regularly interspaced short palindromic repeats.

### 3.5 Comparison of deduced amino acid sequences of NetB between CP280 and strains from avian necrotic enteritis cases

A total of 39 NetB sequences of type G strains isolated from necrotic enteritis cases in chickens and turkeys were obtained from the NCBI database (Supplementary Tables 1, 5). NetB of the 39 strains and CP280 were classified into two types, NetB-1 and NetB-2, based on their deduced amino acid sequences. NetB-1 and NetB-2 were 322 amino acids in length, and their sequences were almost identical. However, the 168th amino acid residue was different between them (Tryptophan in NetB-1 and Alanine in NetB-2). The NetB of CP280 was of the NetB-1 type (Supplementary Table 5).

### 3.6 Comparison of deduced amino acid sequences of CPE between CP280 and strains from human food poisoning cases

The genome sequences of 25 strains with the *cpe* gene isolated from food poisoning cases and one strain reported to have the functional *cpe* gene were available in the NCBI database, 24 of which were type F and two were type E (Supplementary Tables 1, 6). The CPE of the 26 strains were divided into two types: CPE-1 possessed by the 24 type F strains and CPE-2 possessed by the two type E strains. CPE-1 and CPE-2 were 319 and 325 amino acids in length, respectively, and the CPE of CP280 was of the CPE-1 type. The deduced amino acid sequences of CPE were identical for each type; however, there were 18 amino acid differences between the two types (Supplementary Table 6).

## 4 Discussion

In the present study, we reported the first genomic analysis of a *C. perfringens* strain “CP280,” which carries two of the virulence factor genes, *cpe* and *netB*, and revealed the phylogenetic relationships between CP280 and other *C. perfringens* strains in the population.

The fact that CP280 contained *netB* and *cpe* on two different plasmids, pCP280-82k and pCP280-55k, respectively, led to two hypotheses. The first hypothesis was that CP280 was originally a type G strain possessing the *plc/cpa* and *netB* genes and then obtained the *cpe*-positive plasmid pCP280-55k. Second was that CP280 was originally a type F strain carrying the *plc/cpa* and *cpe* genes and subsequently acquired the *netB*-positive plasmid pCP280-82k. Both MLST and genomic analyses performed in the present study revealed that CP280 is genetically closely related to type G strains, strongly supporting the first hypothesis. However, it is unknown where and how the CP280 strain acquired pCP280-55k, since neither type F strains nor any of the other toxinotype strains (types C, D, and E) that may carry the *cpe* gene were isolated from its bovine intestinal content samples. Of note, the deduced amino acid sequence of NetB in CP280 was identical to those of type G strains isolated from chickens with necrotic enteritis. This, along with the close relationship and similar virulence-related gene profiles between CP280 and other type G strains, implies the potential of CP280 to cause avian necrotic enteritis.

Isolation of type G strains from cattle is very rare, and to our knowledge, only one case has been reported previously (Smyth and Martin, 2010); therefore, CP280 was the second *netB*-positive strain isolated from cattle. In addition to the three major toxin genes (*plc*/*cpa*, *netB*, and *cpe*), CP280 harbored 13 additional virulence-related genes. Eleven genes (*pfoA*, *nanH*, *nanI*, *nanJ*, *nanK*, *nagH*, *nagI*, *nagJ*, *nagK*, *colA*, and *ccp*/*cloSI*) were located on the chromosome, and two (two copies of *cpb2*) were located on different plasmids, pCP280-76k and pCP280-55k. In this case, no pathogens other than *C. perfringens* were isolated from the specimens under the conditions tested, while *C. perfringens* was detected at a high concentration (>1.0 × 10^6^ cells/g) from the intestinal contents of the dead cow, and five other type A strains with various STs were also isolated. However, the genomes of the type A strains have not been analyzed, and the virulence-related genes they possess or their phylogenetic relationships with other *C. perfringens* strains are still unknown. It is also unknown whether CP280 was dominant in the *C. perfringens* population in the intestine of the affected cow. Furthermore, since we have attempted to isolate bacteria from specimens only on limited media and under limited conditions, we cannot rule out the possibility that the pathogen responsible for the true cause of the cow’s death has been overlooked. Therefore, it is not possible to determine the involvement of CP280 in the symptoms and lesions observed in the present bovine case. Of note, *cpb2*, present in CP280 with two copies, has been reported to be associated with enteric diseases in domestic animals. Especially in piglets, a strong correlation between the prevalence of *cpb2* in isolates from piglets with enteritis and the absence of *cpb2* in isolates from healthy piglets was reported (Bueschel et al., 2003; Garmory et al., 2000). Therefore, the *cpb2* genes in CP280 may have been involved in the blood-like intestinal contents in the present case, and CP280 may also have the potential to cause enteritis in piglets, although further study is needed to test these hypotheses.

Food poisoning by *C. perfringens* is one of the notable foodborne diseases worldwide, and its symptoms are attributed to CPE encoded by the *cpe* gene. Under the current toxinotyping scheme, type F strains always have the *cpe* gene, and strains of the other three toxinotypes (types C, D, and E) may possess the *cpe* gene. Among the strains analyzed in this study, *cpe* genes were found in the genome sequences of some type C (2/19 strains), type D (5/31 strains), and type E (6/8 strains) strains. The main toxinotype isolated from human food poisoning cases is type F. However, among the 14 *cpe*-positive type C, D, and E strains, a type E strain, a515.17, was also isolated from a human with food poisoning. Therefore, in addition to type F, at least *cpe*-positive type E strains should also be considered as a cause of food poisoning.

Type F strains from human food poisoning cases belonged to BAPS groups 2 and 3, whereas type F strains from affected animals were clustered in BAPS groups 1 and 7, despite being of the same toxinotype. Therefore, the presence of the *cpe* gene alone may be insufficient to cause food poisoning in humans, and other factors may also be necessary to cause the disease. Alternatively, the *cpe* gene of the type F strains in BAPS groups 1 and 7 may be a silent gene and may have lost the ability to produce CPE. CP280 was classified as BAPS group 1 under the datasets and conditions employed in the present study. The BAPS clustering depends not only strongly on the analyzed data set, but also on how the sequence matrix and alignment are constructed. Therefore, strains would not necessarily be classified in the same way as in this study if analyzed using different datasets and conditions. However, the fact that CP280 was classified as BAPS group 1 may suggest that this strain is unlikely to cause food poisoning. In the previous study on the location of the *cpe* gene in the *C. perfringens* genomes, all type F strains carrying *cpe* on their chromosome (i.e., c-*cpe* strains) belonged to the same lineage (corresponding to BAPS group 3 in the present study), and these strains were isolated from food or stool samples associated with food poisoning cases (Jaakkola et al., 2021). In addition, the c-*cpe* strains tended to have fewer virulence-related genes than strains carrying *cpe* on plasmids (i.e., p-*cpe* strains) (Jaakkola et al., 2021). Furthermore, Abdel-Glil et al. (2011) also reported the presence of a lineage (phylogroup I in their study) mainly consisting of c-*cpe* strains involved in human food poisoning cases, and strains in this phylogroup carried less virulence gene homologs than those in the other phylogroups. CP280 was a p-*cpe* strain, carrying a number of virulence-associated genes, and its profile differed significantly from those of BAPS group 3 strains associated with food poisoning cases. These facts may also support the low risk of CP280 causing food poisoning. However, other factors involved in causing food poisoning are unknown. In addition, this strain encodes a CPE identical in deduced amino acid sequence to that of most type F strains from food poisoning cases in humans. Therefore, further study will be needed to determine if CP280 should be considered as a threat not only to animal diseases but also to foodborne diseases in humans.

For further studies to confirm the pathogenicity and virulence of CP280 in chickens, humans, and cattle, challenge studies of CP280 and existing type F and type G strains using animal models and investigation of the expression levels and co-expression of NetB and CPE in different hosts and under different environmental conditions will be needed. Studies on the cytotoxicity of CPE and NetB of CP280 on different host cells, synergistic effects of the two toxins in the host, regulatory mechanisms of toxin expression at the molecular level, and host factors that control the expression will also provide useful information in understanding the role of these toxins in the hosts. To our knowledge, other than CP280, two strains with both *cpe* and *netB* have been reported, both isolated in Germany (Gad et al., 2011). Since the genomes of these German strains have not been analyzed, it is not known whether they are also derived from type G strains like CP280. Studies before the current seven toxinotypes (Rood et al., 2018) were proposed did not necessarily confirm the presence of *cpe* and *netB*; therefore, strains with these two genes could be present but have been undetected. To understand whether CP280 is an isolated case or part of an emerging trend, it is important to analyze the genomes of the German strains and reveal the locations of *cpe* and *netB* on their genomes and the history of their toxin gene acquisition. In addition, large-scale surveillance will need to be conducted, not only in Japan but around the world, including re-examination of strains classified as type A in the past and active detection of *cpe*+/*netB*+ strains in food and animal products. Furthermore, examining the stability of *cpe* and *netB* on plasmids, the stability of the *netB*-positive and *cpe*-positive plasmids in CP280, and the ability of these plasmids to horizontally transfer to other *C. perfringens* strains would also be helpful in predicting whether strains like CP280 could spread around the world in the future and become an animal and public health threat.

Before starting the genetic analyses of CP280, there were three options as to which toxinotype the *cpe*+/*netB*+ strain CP280 should be classified; that is, type F, type G, or a new toxinotype. Since this study revealed that CP280 is most likely derived from type G, we propose to assign CP280 as type G and amend the condition for *cpe* gene possession of this toxinotype from “−” to “+/−” (Table 5). We do not believe that our proposal will cause confusion in *C. perfringens* research since the *cpe* gene possession condition is also “+/−” for types C, D, and E in the current toxinotyping scheme. As current PCR assays targeting the six major toxin genes (e.g., the multiplex PCR reported by Rood et al., 2018) will not miss strains carrying *netB* and *cpe*, we also do not believe that our proposed typing scheme will cause significant confusion in the future diagnosis and surveillance of *C. perfringens* outbreaks in animals and humans. However, it will be necessary to consider that some type G strains may have the potential to cause both necrotic enteritis in chickens and food poisoning in humans. In addition, if the *netB*-positive and *cpe*-positive plasmids are unstable in *C. perfringens*, these plasmids could be lost from the bacteria during isolation from the specimen. In such cases, *cpe*-negative type G strains could be isolated from human food poisoning cases and *netB*-negative type F strains could be isolated from poultry necrotic enteritis cases. We believe that our present study and future studies on plasmid stability will help us understand the true causes of these irregular cases and lead to a better understanding of the epidemiology of *C. perfringens* outbreaks in both animals and humans.

**Table 5:** Amended toxin-based typing scheme of *Clostridium perfringens* proposed in the present study.

Toxinotype	Toxin (gene)					
	Alpha-toxin	Beta-toxin	Epsilon-toxin	Iota-toxin	Enterotoxin	NetB
	(*plc* or *cpa*)	(*cpb*)	(*etx*)	(*iap* and *ibp*)	(*cpe*)	(*netB*)
A	+	−	−	−	−	−
B	+	+	+	−	−	−
C	+	+	−	−	±	−
D	+	−	+	−	±	−
E	+	−	−	+	±	−
F	+	−	−	−	+	−
G	+	−	−	−	±	+

The amendment to the typing requirement proposed in the present study is highlighted in the pink cell, and the toxinotype and toxin gene associated with this amendment are highlighted in yellow cells.

## Data Availability

Allele profiles of *C. perfringens* strains CP280-CP285 by the MLST analysis were deposited in the PubMLST database (https://pubmlst.org/bigsdb?db=pubmlst_cperfringens_isolates) as id 983-988, respectively. Sequenced raw-read data from MinION and DNBSEQ-G400 platforms and genetic information of the *C. perfringens* strain CP280 were deposited in the DDBJ/GenBank/EMBL database under the BioProject accession number PRJDB16159, BioSample accession number SAMD00628698, and DRA accession number DRA016639. The complete genome sequence data for *C. perfringens* strain CP280 were also deposited in the DDBJ/GenBank/EMBL database with the accession numbers AP028647-AP028653.

## Footnotes

^1^ https://pubmlst.org/organisms/clostridium-perfringens

^2^ https://ftp.ncbi.nlm.nih.gov/genomes/all/GCF/

^3^ https://cagecat.bioinformatics.nl

^4^ https://pubmlst.org

## References

[ref1] Abdel-Glil M. Y. Thomas P. Linde J. Busch A. Wieler L. H. Neubauer H.. (2011). Comparative in silico genome analysis of *Clostridium perfringens* unravels stable phylogroups with different genome characteristics and pathogenic potential. Sci. Rep. 11:6756. doi: 10.1038/s41598-021-86148-8, PMID: 33762628 PMC7991664

[ref2] Al-Sheikhly F. Truscott R. B. (1977). The interaction of *Clostridium perfringens* and its toxins in the production of necrotic enteritis of chickens. Avian Dis. 21, 256–263. doi: 10.2307/1589345, PMID: 194572

[ref3] Brynestad S. Granum P. E. (1999). Evidence that Tn*5565*, which includes the enterotoxin gene in *Clostridium perfringens*, can have a circular form which may be a transposition intermediate. FEMS Microbiol. Lett. 170, 281–286. doi: 10.1111/j.1574-6968.1999.tb13385.x, PMID: 9919679

[ref4] Brynestad S. Synstad B. Granum P. E. (1997). The *Clostridium perfringens* enterotoxin gene is on a transposable element in type a human food poisoning strains. Microbiology 143, 2109–2115. doi: 10.1099/00221287-143-7-2109, PMID: 9245800

[ref5] Bueschel D. M. Jost B. H. Billington S. J. Trinh H. T. Songer J. G. (2003). Prevalence of *cpb2*, encoding beta2 toxin, in *Clostridium perfringens* field isolates: correlation of genotype with phenotype. Vet. Microbiol. 94, 121–129. doi: 10.1016/s0378-1135(03)00081-6, PMID: 12781480

[ref6] Camacho C. Coulouris G. Avagyan V. Ma N. Papadopoulos J. Bealer K.. (2009). BLAST+: architecture and applications. BMC Bioinformatics 10:421. doi: 10.1186/1471-2105-10-421, PMID: 20003500 PMC2803857

[ref7] Chen Y. Chen Y. Shi C. Huang Z. Zhang Y. Li S.. (2018). SOAPnuke: a MapReduce acceleration-supported software for integrated quality control and preprocessing of high-throughput sequencing data. Gigascience 7, 1–6. doi: 10.1093/gigascience/gix120 PMC5788068 29220494

[ref8] Cornillot E. Saintjoanis B. Daube G. Katayama S. Gran-um P. E. Canard B.. (1995). The enterotoxin gene (*cpe*) of *Clostridium perfringens* can be chromosomal or plasmid-borne. Mol. Microbiol. 15, 639–647. doi: 10.1111/j.1365-2958.1995.tb02373.x, PMID: 7783636

[ref9] De Coster W. D’Hert S. Schultz D. T. Cruts M. Broeckhoven C. V. (2018). NanoPack: visualizing and processing long-read sequencing data. Bioinformatics 34, 2666–2669. doi: 10.1093/bioinformatics/bty149 29547981 PMC6061794

[ref10] Deguchi A. Miyamoto K. Kuwahara T. Miki Y. Kaneko I. Li J.. (2009). Genetic characterization of type a enterotoxigenic *Clostridium perfringens* strains. PLoS One 4:e5598:e5598. doi: 10.1371/journal.pone.0005598, PMID: 19479065 PMC2682570

[ref11] Felsenstein J. (1985). Confidence limits on phylogenies: an approach using the bootstrap. Evolution 39, 783–791. doi: 10.2307/2408678, PMID: 28561359

[ref12] Gad R. Hauck R. Krüger M. Hafez H. M. (2011). Prevalence of *Clostridium perfringens* in commercial Turkey and layer flocks. Arch.Geflügelk. 75, 74–79. doi: 10.1016/s0003-9098(25)00758-1, PMID: 40224509

[ref13] Garmory H. S. Chanter N. French N. P. Bueschel D. Songer J. G. Titball R. W. (2000). Occurrence of *Clostridium perfringens* β2-toxin amongst animals, determined using genotyping and subtyping PCR assays. Epidemiol. Infect. 124, 61–67. doi: 10.1017/s0950268899003295, PMID: 10722131 PMC2810884

[ref14] Gilchrist C. L. M. Chooi Y.-H. (2021). Clinker & clustermap.Js: automatic generation of gene cluster comparison figures. Bioinformatics 37, 2473–2475. doi: 10.1093/bioinformatics/btab007 33459763

[ref15] Gohari I. M. Kropinski A. M. Weese S. J. Parreira V. R. Whitehead A. E. Boerlin P.. (2016). Plasmid characterization and chromosome analysis of two *netF*+ *Clostridium perfringens* isolates associated with foal and canine necrotizing enteritis. PLoS One 11:e0148344. doi: 10.1371/journal.pone.0148344, PMID: 26859667 PMC4747519

[ref16] Goris J. Konstantinidis K. T. Klappenbach J. A. Coenye T. Vandamme P. Tiedje J. M. (2007). DNA-DNA hybridization values and their relationship to whole-genome sequence similarities. Int. J. Syst. Evol. Microbiol. 57, 81–91. doi: 10.1099/ijs.0.64483-0, PMID: 17220447

[ref17] Islam A. A. Nakatani M. Nakajima T. Kohda T. Mukamoto M. (2021). The cytotoxicity and molecular mechanisms of the *Clostridium perfringens* NetB toxin. J. Vet. Med. Sci. 83, 187–194. doi: 10.1292/jvms.20-0623, PMID: 33342969 PMC7972886

[ref18] Jaakkola K. Virtanen K. Lahti P. Keto-Timonen R. Lindström M. Korkeala H. (2021). Comparative genome analysis and spore heat resistance assay reveal a new component to population structure and genome epidemiology within *Clostridium perfringens* enterotoxin-carrying isolates. Front. Microbiol. 12:717176. doi: 10.3389/fmicb.2021.717176, PMID: 34566921 PMC8456093

[ref19] Jolley K. A. Bray J. E. Maiden M. C. (2018). Open-access bacterial population genomics: BIGSdb software, the PubMLST.org website and their applications. Wellcome Open Res. 3:124. doi: 10.12688/wellcomeopenres.14826.1, PMID: 30345391 PMC6192448

[ref20] Keyburn A. L. Boyce J. D. Vaz P. Bannam T. L. Ford M. E. Parker D.. (2008). NetB, a new toxin that is associated with avian necrotic enteritis caused by *Clostridium perfringens*. PLoS Pathog. 4:e26. doi: 10.1371/journal.ppat.0040026, PMID: 18266469 PMC2233674

[ref21] Keyburn A. L. Sheedy S. A. Ford M. E. Williamson M. M. Awad M. M. Rood J. I.. (2006). Alpha-toxin of *Clostridium perfringens* is not an essential virulence factor in necrotic enteritis in chickens. Infect. Immun. 74, 6496–6500. doi: 10.1128/IAI.00806-06, PMID: 16923791 PMC1695520

[ref22] Keyburn A. L. Yan X.-X. Bannam T. L. Immerseel F. V. Rood J. I. Moore R. J. (2010). Association between avian necrotic enteritis and *Clostridium perfringens* strains expressing NetB toxin. Vet. Res. 41:21. doi: 10.1051/vetres/2009069, PMID: 19931005 PMC2797654

[ref23] Kiu R. Hall L. J. (2018). An update on the human and animal enteric pathogen *Clostridium perfringens*. Emerg. Microbes Infect. 7:141. doi: 10.1038/s41426-018-0144-8, PMID: 30082713 PMC6079034

[ref24] Kurtz S. Phillippy A. Delcher A. L. Smoot M. Shumway M. Antonescu C.. (2004). Versatile and open software for comparing large genomes. Genome Biol. 5:R12. doi: 10.1186/gb-2004-5-2-r12, PMID: 14759262 PMC395750

[ref25] Lacey J. A. Allnutt T. R. Vezina B. Van T. T. H. Stent T. Han X.. (2018). Whole genome analysis reveals the diversity and evolutionary relationships between necrotic enteritis-causing strains of *Clostridium perfringens*. BMC Genomics 19:379. doi: 10.1186/s12864-018-4771-1, PMID: 29788909 PMC5964661

[ref26] Letunic I. Bork P. (2021). Interactive tree of life (iTOL) v5: an online tool for phylogenetic tree display and annotation. Nucleic Acids Res. 49, W293–W296. doi: 10.1093/nar/gkab301, PMID: 33885785 PMC8265157

[ref27] Li J. Adams V. Bannam T. L. Miyamoko K. Garcia J. P. Uzal F. A.. (2013). Toxin plasmids of *Clostridium perfringens*. Microbiol. Mol. Biol. Rev. 77, 208–233. doi: 10.1128/MMBR.00062-12, PMID: 23699255 PMC3668675

[ref28] Mada T. Goto Y. Kumagai M. Sakai H. Kanamori H. Takamatsu D. (2023). A calf with hind limb paralysis and dysstasia and a genome sequence analysis of an isolated *Clostridium perfringens* toxinotype E strain. J. Vet. Med. Sci. 85, 279–289. doi: 10.1292/jvms.22-0432, PMID: 36653149 PMC10076203

[ref29] Matches J. R. Liston J. Curran D. (1974). *Clostridium perfringens* in the environment. Appl. Microbiol. 28, 655–660. doi: 10.1128/am.28.4.655-660.1974, PMID: 4371684 PMC186792

[ref30] Miyamoto K. Fisher D. J. Li J. Sayeed S. Akimoto S. McClane B. A. (2006). Complete sequencing and diversity analysis of the enterotoxin-encoding plasmids in *Clostridium perfringens* type a non-food-borne human gastrointestinal disease isolates. J. Bacteriol. 188, 1585–1598. doi: 10.1128/JB.188.4.1585-1598.2006, PMID: 16452442 PMC1367241

[ref31] Miyamoto K. Yumine N. Mimura K. Nagahama M. Li J. McClane B. A.. (2011). Identification of novel *Clostridium perfringens* type E strains that carry an iota toxin plasmid with a functional enterotoxin gene. PLoS One 6:e20376. doi: 10.1371/journal.pone.0020376, PMID: 21655254 PMC3105049

[ref32] Nauerby B. Pendersen K. Madsen M. (2003). Analysis by pulsed-field gel electrophoresis of the genetic diversity among *Clostridium perfringens* isolates from chickens. Vet. Microbiol. 94, 257–266. doi: 10.1016/s0378-1135(03)00118-4, PMID: 12814893

[ref33] Okamoto M. Kumagai M. Kanamori H. Takamatsu D. (2021). Antimicrobial resistance genes in bacteria isolated from japanese honey, and their potential for conferring macrolide and lincosamide resistance in the american foulbrood pathogen *Paenibacillus larvae*. Front. Microbiol. 12:667096. doi: 10.3389/fmicb.2021.667096, PMID: 33995331 PMC8116656

[ref34] Page A. J. Cummins C. A. Hunt M. Wong V. K. Reuter S. Holden M. T. G.. (2015). Roary: rapid large-scale prokaryote pan genome analysis. Bioinformatics 31, 3691–3693. doi: 10.1093/bioinformatics/btv421, PMID: 26198102 PMC4817141

[ref35] Page A. J. Taylor B. Delaney A. J. Soares J. Seemann T. Keane J. A.. (2016). SNP-sites: rapid efficient extraction of SNPs from multi-FASTA alignments. Microb. Genom. 2:e000056. doi: 10.1099/mgen.0.000056, PMID: 28348851 PMC5320690

[ref36] Parreira V. R. Costa M. Eikmeyer F. Bolm J. Prescott J. F. (2012). Sequence of two plasmids from *Clostridium perfringens* chicken necrotic enteritis isolates and comparison with *C. perfringens* conjugative plasmids. PLoS One 7:e49753. doi: 10.1371/journal.pone.0049753, PMID: 23189158 PMC3506638

[ref37] Pritchard L. Glover R. H. Humphris S. Elphinstone J. G. Toth L. K. (2016). Genomics and taxonomy in diagnostics for food security: soft-rotting enterobacterial plant pathogens. Anal. Methods 8, 12–24. doi: 10.1039/C5AY02550H

[ref38] Profeta F. Di Giannatale E. Orsini M. Ancora M. Smoglica C. Cammà C.. (2020). Draft genome sequence of *Clostridium perfringens netB*-positive strain 2016TE7641_69, isolated from the intestine of a diseased Turkey in Italy. Microbiol. Resour. Announc. 9, e00065–e00020. doi: 10.1128/MRA.00065-20, PMID: 32409527 PMC7225526

[ref39] Ronco T. Stegger M. Ng K. L. Lilje B. Lyhs U. Andersen P. S. (2017). Genome analysis of *Clostridium perfringens* isolates from healthy and necrotic enteritis infected chickens and turkeys. BMC. Res. Notes 10:270. doi: 10.1186/s13104-017-2594-9, PMID: 28693615 PMC5504799

[ref40] Rood J. I. Adams V. Lacey J. Lyras D. McClane B. A. Melville S. B.. (2018). Expansion of the *Clostridium perfringens* toxin-based typing scheme. Anaerobe 53, 5–10. doi: 10.1016/j.anaerobe.2018.04.011, PMID: 29866424 PMC6195859

[ref41] Saito N. Nei M. (1987). The neighbor-joining method: a new method for reconstructing phylogenetic trees. Mol. Biol. Evol. 4, 406–425. doi: 10.1093/oxfordjournals.molbev.a040454, PMID: 3447015

[ref42] Shen W. Le S. Hu F. (2016). SeqKit: a cross-platform and ultrafast toolkit for FASTA/Q file manipulation. PLoS One 11:e0163962. doi: 10.1371/journal.pone.0163962, PMID: 27706213 PMC5051824

[ref43] Smyth J. A. Martin T. G. (2010). Disease producing capability of *netB* positive isolates of *C. perfringens* recovered from normal chickens and a cow, and *netB* positive and negative isolates from chickens with necrotic enteritis. Vet. Microbiol. 146, 76–84. doi: 10.1016/j.vetmic.2010.04.022, PMID: 20537820

[ref44] Stecher G. Tamura K. Kumar S. (2020). Molecular evolutionary genetics analysis (MEGA) for macOS. Mol. Biol. Evol. 37, 1237–1239. doi: 10.1093/molbev/msz312, PMID: 31904846 PMC7086165

[ref45] Tamura K. Nei M. Kumar S. (2004). Prospects for inferring very large phylogenetic by using the neighbor-joining method. Proc. Natl. Acad. Sci. USA 101, 11030–11035. doi: 10.1073/pnas.0404206101, PMID: 15258291 PMC491989

[ref46] Tamura K. Stecher G. Kumar S. (2021). MEGA 11: molecular evolutionary genetics analysis version 11. Mol. Biol. Evol. 38, 3022–3027. doi: 10.1093/molbev/msab120, PMID: 33892491 PMC8233496

[ref47] Tanizawa Y. Fujisawa T. Nakamura Y. (2018). DFAST: a flexible prokaryotic genome annotation pipeline for faster genome publication. Bioinformatics 34, 1037–1039. doi: 10.1093/bioinformatics/btx713, PMID: 29106469 PMC5860143

[ref48] Tonkin-Hill G. Lees J. A. Bentley S. D. Frost S. D. W. Corander J. (2018). RhierBAPS: an R implementation of the population clustering algorithm herBAPS. Wellcome Open Res. 3:93. doi: 10.12688/wellcomeopenres.14694.1, PMID: 30345380 PMC6178908

[ref49] Van den Belt M. Gilchrist C. Booth T. J. Chooi Y.-H. Medema M. H. Alanjary M. (2023). CAGECAT: the comparative gene cluster analysis toolbox for rapid search and visualization of homologous gene clusters. BMC Bioinformatics 24:181. doi: 10.1186/s12859-023-05311-2, PMID: 37131131 PMC10155394

[ref50] Walker B. J. Abeel T. Shea T. Priest M. Abouelliel A. Sakthikumar S.. (2014). Pilon: an integrated tool for comprehensive microbial variant detection and genome assembly improvement. PLoS One 9:e112963. doi: 10.1371/journal.pone.0112963, PMID: 25409509 PMC4237348

[ref51] Wick R. R. Judd L. M. Gorrie C. L. Holt K. E. (2017). Unicycler: resolving bacterial genome assemblies from short and long sequencing reads. PLoS Comput. Biol. 13:e1005595. doi: 10.1371/journal.pcbi.1005595, PMID: 28594827 PMC5481147

